# Circulating levels of the anti-oxidant indoleproprionic acid are associated with higher gut microbiome diversity

**DOI:** 10.1080/19490976.2019.1586038

**Published:** 2019-04-29

**Authors:** Cristina Menni, Marisa Matey Hernandez, Marius Vital, Robert P. Mohney, Tim D. Spector, Ana M. Valdes

**Affiliations:** a Department of Twin Research and Genetic Epidemiology, King’s College London, London, UK; b Microbial Interactions and Processes, Helmholtz Centre for Infection Research, Braunschweig, Germany; c Discovery and Translational Sciences, Metabolon Inc, Raleigh-Durham, NC, USA; d MSK Theme, NIHR Nottingham Biomedical Research Centre, Nottingham, UK; e School of Medicine, Nottingham City Hospital, Nottingham, UK

**Keywords:** Indoleproprionate, microbiome diversity, metabolic syndrome

## Abstract

The gut microbiome has recently emerged as an important regulator of insulin resistance and abdominal obesity. The tryptophan metabolite generated by the gut microbiome, indoleproprionic acid (IPA) has been shown to predict the onset of type 2 diabetes. IPA is a metabolite produced by gut microbes from dietary tryptophan that exhibits a high degree of inter-individual variation. The microbiome composition parameters that are associated with circulating levels of this potent anti-oxidant have however not been investigated to date in human populations. In 1018 middle-aged women from the TwinsUK cohort, we assessed the relationship between serum IPA levels and gut microbiome composition targeting the 16S rRNA gene. Microbiome alpha-diversity was positively correlated with serum indoleproprionic acid levels (Shannon Diversity: Beta[95%CI] = 0.19[0.13;0.25], P = 6.41 × 10^−10^) after adjustment for covariates. Sixteen taxa and 12 operational taxonomic units (OTUs) associated with IPA serum levels. Among these are positive correlations with the butyrate-producing *Faecalibacterium prausnitzii*, the class Mollicutes and the order RF39 of the *Tenericutes*, and *Coprococcus* Negative correlations instead were observed with *Eubacterium dolichum* previously shown to correlate with visceral fat mass and several genera in the Lachnospiraceae family such as Blautia and Ruminococcus previously shown to correlate with obesity. Microbiome composition parameters explained ~20% of the variation in circulating levels of IPA, whereas nutritional and host genetic parameters explained only ~4%. Our data confirm an association between IPA circulating levels and metabolic syndrome parameters and indicate that gut microbiome composition influences IPA levels.

## Introduction

Indoleproprionic acid (IPA) is an antioxidant predictive of a lower risk of developing type 2 diabetes (T2D).^1^ Indeed, De Mello et al.^1^ compared IPA levels in individuals with impaired glucose tolerance, some of whom developed T2D over 15 years. Higher IPA levels are associated with reduced likelihood of developing T2D and this was further replicated in a Swedish population.^1^

Indolepropionic acid is a deamination product of the amino acid tryptophan. It accumulates in human serum, exhibits a high degree of inter-individual variation^2^ and regulates gastrointestinal barrier function via its interaction with the pregnane X receptor (PXR).^3^

Dietary and genetic factors have been implicated in the circulating levels of this compound in humans, and the gut microbes linked to IPA have been studied in mice.

In terms of dietary factors, IPA has been reported to be strongly associated with intake of dietary fibre^4^ which is known to correlate with higher microbiome diversity and higher production of short-chain fatty acids (SCFAs).^5^ Therefore it might be argued that the effect of this compound might be reflecting simply the beneficial effect of fiber on insulin resistance and fatty acid metabolism in general. Circulating levels of IPA appear to have a heritable component and have been shown to be associated with genetic variants in large metabolomic screens.^6^

In mice, production of IPA has been shown to be completely dependent on the presence of gut microbiome and to be specifically related to the bacterium *Clostridium sporogenes*^6^. More recently, Dodd and collaborators^7^ cultivated 36 bacterial isolates in the same tryptophan-containing medium and found that 5 other bacterial isolates (*Peptostreptooccus anaerobius*, CC14N and three strains of *Clostridum cadaveris*) were able to produce IPA. If higher levels of this compound have health benefits, as suggested by the literature, then understanding the relative contribution of the gut microbiome to its levels would become relevant in order to understand how to increase its production in the gut. However, the gut microbiome components linked to higher or lower levels of this compound have not been studied in human populations as yet nor has the relative importance of gut microbiome factors relative to dietary or genetic factors.

We hypothesized that gut microbiome and dietary intake of tryptophan would have the strongest influence on levels of IPA. In this study, we have therefore quantified the contribution of microbiome composition, dietary fiber and tryptophan intake and genetic variation in humans to circulating levels of IPA in a large cohort of twins.

### Results

The descriptive characteristics of the study participants are presented in Table 1. One thousand eighteen females with concurrent IPA and microbiome data were included in the analysis.

**Table 1.** Descriptive characteristics of the study population.

Phenotype	*N*	*%*
N	1018	
Females	1018	100
	***Mean***	***SD***
Age, yrs	65.62	7.72
BMI, Kg/m2	26.25	4.90
Fibre intake, gr	20.92	7.21
HOMA2-IR	0.93	0.67
*Indices of microbiome diversity*		
Shannon Diversity	5.14	0.69
Simpson Diversity	0.92	0.06
CHAO1	812.59	331.27
Number of observed OTUs	338.85	89.27

**Figure 1.**
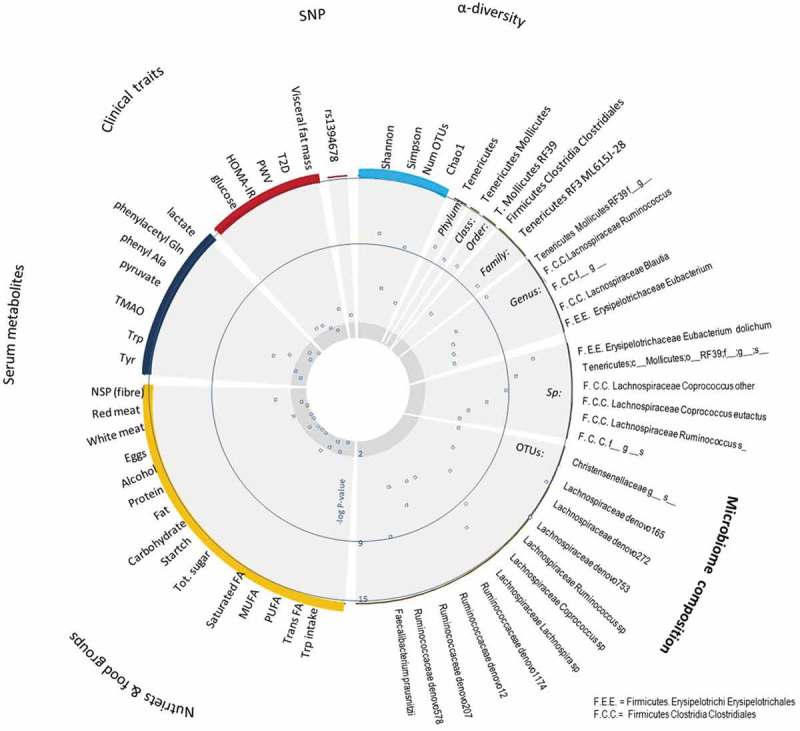
CIRCOS plot showing the association (-log10 p-value) with indeloproprionic acid (IPA) levels and clinical parameters, genetic parameters, dietary and food intake parameters and microbiome parameters, including four measures of alpha diversity.

We first tested whether IPA levels were associated with clinical parameters related to metabolic syndrome in our cohort and we find that indeed it is strongly negatively associated with visceral fat (Beta (SE) = −0.057 (0.018), P = 0.002), insulin resistance (Beta (SE) = −0.046 (0.019), P = 0.014), arterial stiffness (Beta (SE) = −0.128 (0.064), P = 0.045), and fasting glucose (Beta (SE) = −0.08 (0.032, P = 0.012) (Table S1, Figure 1). We then investigated what are the factors contributing to circulating levels of IPA. To do this we considered three sources: genetics, diet and gut microbiome composition.

As part of the analysis, we also included genetic variation known to influence levels of IPA^8^ mapping specifically to the *ACSM2A* gene. Although several SNPs map to the genetic region known to be associated with IPA levels, they are all highly correlated with each other and therefore we concentrated on only the SNP with the strongest p-value, namely rs1394678 (Figure 1).

IPA is derived from tryptophan so we have considered various dietary sources of tryptophan as contributors to its circulating levels (Figure 1).

When we assessed the relationship between circulating levels of IPA and four different measures of microbiome alpha diversity (Table 2) we found that it was positively correlated with all four measures of alpha-diversity, and these associations remained significant even after adjusting for dietary fiber intake. This suggests that the association between the gut microbiome and IPA levels is not confounded by fiber intake.

**Table 2.** Association between circulating IPA levels and alpha diversity measures.

Diversity	Beta[95%CI]	P
Shannon	0.19[0.13;0.25]	6.41 × 10^−10^
Simpson	0.13[0.07;0.18]	2.92 × 10^−5^
Number of observed OTUs	0.18[0.12;0.24]	3.22 × 10^−9^
Chao1	0.11[0.05;0.17]	2.07 × 10^−4^

**Figure 2.**
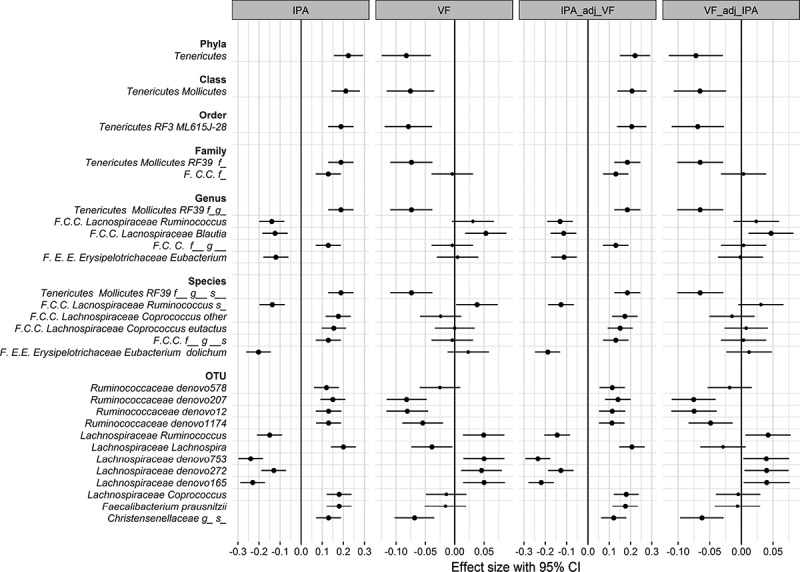
Forest plot showing an association between bacterial taxa and OTUs and IPA circulating levels and visceral fat mass.

We identified 16 taxa (Table S2) and 56 operational taxonomic units (OTUs) (Table S3) associated with IPA serum levels. The strongest associations seen were positive associations with *RF39* assigned to the *Tenericutes* phylum and negative associations with OTUs from the *Lachnospiraceae* family. We also found positive correlations with the butyrate-producing *Faecalibacterium prausnitzii*, the genus *Coprococcus*,^9^ the class *Mollicutes* and several OTUs affiliated with the *Ruminococcaceae* as well as one from the family *Chistensenellaceae*. Negative correlations instead were observed with several *Eubacterium* species including *E. dolichum* that has been previously reported to be associated with increased visceral fat (Figure 1).^10^ When we included the 56 OTUs in a stepwise linear regression model, we found a panel of 12 OTUs independently associated with IPA levels after adjusting for covariates (Figures 1 and 2).

In terms of relative abundances, we found that by far the most common taxon positively correlated with IPA levels are unannotated sequences within the order *Clostridiales* not assigned a lower taxonomy with an average relative abundance of almost 10% (Table S2), followed by the genus *Coprococcus* (1.91%) and bacteria within the order *Mollicutes RF39* (1.71%) of the phylum *Tenericutes*. In terms of negative correlations, the most common taxon associated with lower levels of IPA was the genus *Blautia* of the phylum *Firmicutes.*

**Figure 3.**
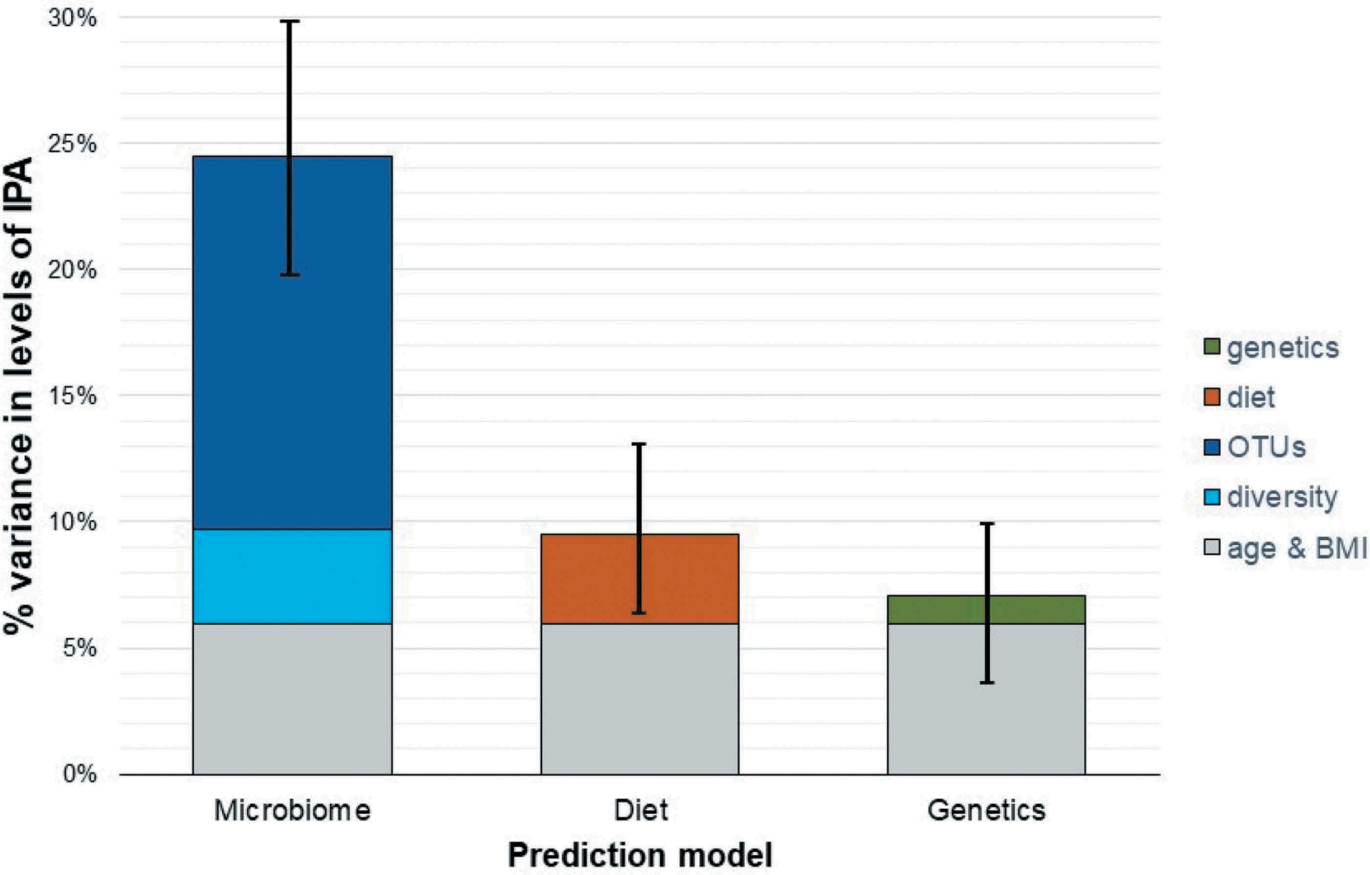
Proportion of the variance in IPA circulating levels explained by microbiome parameters, dietary intake parameters and genetic parameters in 1018 women from the TwinsUK cohort.

We then quantified how much these various factors contributed quantitatively and found that age and BMI contribute 5.97% of the variance in IPA levels. Dietary intake parameters (fiber intake, saturated fats, and eggs) contributed an additional 3.51% of the variance (Figure 3), the genetic variant contributed 1.1% of the variance, gut-microbiome alpha diversity 3.72% and the 12 OTUs independently associated to IPA levels 14.77% (Figure 3). Thus, the strongest contribution to the interindividual variation of IPA levels came from microbiome composition.

Finally, we investigated how much the bacterial OTUs independently associated positively and negatively with circulating IPA and the IPA associated taxa reflect visceral fat mass. Visceral fat is not only an important contributor to metabolic syndrome but was the phenotypic trait most strongly associated in our data (Table S1); 9 out of the 12 independent OTUs and 8 out of the 16 IPA associated taxa were also nominally associated with visceral fat mass after adjusting for age, and BMI (Figure 2). In all cases, we found that those associated negatively with IPA were positively correlated with visceral fat whereas those positively correlated with IPA were negatively correlated with visceral fat (Figure 2). Importantly all the bacterial associations with IPA remained statistically significant after adjusting for visceral fat mass but one out nine associations between visceral fat mass and the bacterial OTUs was no longer statistically significant after adjustment for IPA levels (Figure 2).

## Discussion

In this study we have investigated for the first time in humans, the relationship between IPA circulating levels, gut microbiome composition and metabolic health in 1018 women. In our data, we find that IPA is strongly correlated with high microbiome diversity in spite of the fact that only a handful of bacteria strains are known to be able to produce IPA.^7^ We also confirm that IPA is strongly linked to dietary fiber intake and that higher IPA circulating levels correlate with lower risk of a number of metabolic syndrome parameters as shown by Mello et al.^1^

None of the strains from murine gut^7^, which have been shown to produce IPA, were found in our study to be correlated with IPA production. Most of the strains reported to produce IPA from tryptophan belong to the order *Clostridiales*. In our data, we find a bacteria belonging to that order (>9.8% abundance) strongly correlated with higher levels of IPA. This suggests that the lack of taxonomic resolution of 16S rRNA data hampers accurate detection of the IPA-producing strains. Metagenome-based analyses data will allow for properly measuring IPA producers in human fecal DNA. On the other hand, we find that a number of bacterial OTUs that have been shown not to produce IPA,^7^ such as *Tenericutes, Coprococcus sp* and *Faecalibacterium prasunitzii* are positively correlated with IPA levels. It is possible that these bacteria thrive in a similar environment to that of IPA producers and hence they may be signatures for IPA production even if they lack this function. It is also possible that the bacteria that actually produce IPA thrive in a complex environment characterized by higher diversity.

Previous studies had investigated the genetic variation that contributes to metabolite circulating levels including those of IPA and had reported a genome-wide significant region mapping specifically to the *ACSM2A* gene.^8^ We report that a variant in the *ACSM2A* gene contributes to the overall variation in IPA circulating levels^8^ in addition to dietary and gut microbiome factors. This gene encodes a mitochondrial xenobiotic/medium-chain fatty acid: CoA ligase (ACSM). The glycine conjugation pathway is a two-step enzymatic reaction responsible for the metabolism/detoxification of substrates that include xenobiotics produced by gut microbiome, metabolites from organic acidemias, and medium-chain fatty acids (MCFAs).^11^ The first step is the activation to an acyl-CoA by the ACSMs and the second step is the conjugation to glycine by glycine N-acyltransferase (GLYAT). Therefore, the genetic variants influencing IPA levels are likely to be related to the metabolism and excretion of IPA rather than its production. In fact, the contribution of genetic variation in the *ACSM2A* is fairly modest when compared to that of diet and microbiome composition.

We note some limitations to the current study. The study sample consisted exclusively of adult women and therefore the results may be different in men. The dietary influences have been assessed from food frequency questionnaires and not from food diaries or from controlled dietary interventions. The resolution from the 16S rRNA gene does not allow for strain or in many cases not even species identification. For this reason, the current study cannot provide functional insights into IPA production potential by the microbiome to prove causality. Shotgun-sequencing that provides a deeper taxonomic resolution as well as functional insights appears necessary to properly characterize the bacterial taxa involved in IPA production in the human gut.

Notwithstanding the above limitations, this is the first study to investigate the dietary, genetic and microbiome factors to the circulating levels of this powerful anti-oxidant.

### Subjects and methods

Study subjects were female twins enrolled in the TwinsUK registry, a national register of adult twins recruited as volunteers without selecting for any particular disease or trait traits.^12^ In this study, we analyzed data from 1018 female twins with concurrent 16s microbiome data and serum metabolomics. The study was approved by NRES Committee London–Westminster, and all twins provided informed written consent.

**Type 2 diabetes**. T2D cases were defined as individuals with fasting glucose levels≥7mmol/L at time of initial sampling and at subsequent visits, while T2D “super controls” were defined as subjects with fasting glucose levels between 3.9mmol/L and 5mmol/L, as was done for the original metabolomic study of T2D.^13^

**Visceral fat measurement by DXA**. Estimates of visceral fat mass were derived from DXA measurements of whole body composition as previously described.^14^

**HOMA-IR** – Fasting insulin and glucose levels were measured for the twin cohort using the same methods as previously described.^12^ The homeostasis model assessment-estimated insulin resistance (HOMA-IR) was calculated multiplying overnight fasting plasma insulin (FPI) by overnight fasting plasma glucose (FPG), then dividing by the constant 22.5, i.e. HOMA-IR = (FPI×FPG)/22.515.

**Fibre and fatty acid intake**: A validated 131-item semi-quantitative Food Frequency Questionnaire (FFQ) established for the EPIC (European Prospective Investigations into Cancer and Nutrition)-Norfolk study^15^ was used to assess dietary intake. Estimated intakes of essential fatty acids and fiber (in grams per day) were derived from the UK Nutrient Database^16^ and were adjusted for energy intake using the residual method prior to analysis.^17^

**Genetic variation**: Here we dissected the IPA genome-wide association scan (GWAS) data that was previously generated and published as part of our GWAS–metabolomics study.^8^ GWAS (in the HapMap 2–based imputed genotype data set) was conducted on 6056 individuals from TwinsUK and 1768 from KORA as previously described.^8^ Association results were combined in Metal^18^ using inverse variance meta-analysis based on effect size estimates and standard errors, adjusting for genomic control.

**Indolepropionate**: Circulating IPA levels were measured using ultra-high performance liquid chromatography-tandem mass-spectrometry by the metabolomics provider Metabolon Inc. (Durham, USA) on 1018 fasting serum samples from participants in the TwinsUK study, as described previously.^19^

**Microbiome analysis**: Faecal samples were collected and the composition of the gut microbiome was determined by 16S rRNA gene sequencing carried out as previously described.^20^ Briefly, the V4 region of the 16S rRNA gene was amplified and sequenced on Illumina MiSeq. Reads were then summarised to operational taxonomic units (OTUs) Quality control was carried out on a per sample basis, discarding paired-ends with an overlap of less than 200nt and removing chimeric sequences using de novo chimera detection in USEARCH.^21^ De novo OTU clustering was then carried across all reads using Sumaclust within QIIME 1.9.0, grouping reads with a 97% identity threshold.^22,23^ OTU counts were converted to log-transformed relative abundances, with zero counts handled by the addition of an arbitrary value (10^−6^). The residuals of the OTU abundances were taken from linear models, accounting for technical covariates including sequencing depth, sequencing run, sequencing technician and sample collection method. These residuals were inverse normalized, as they were not normally distributed, and used in downstream analyses. In order to calculate alpha diversity, the complete OTU count table was rarefied to 10,000 sequences per sample 50 times. Alpha diversity metrics were calculated for each sample in each of the rarefied tables and final diversity measures taken as the mean score across all 50. Alpha diversities were quantified as observed OTU counts and Shannon and Simpson diversity indices. Alpha diversity indexes were standardized to have mean 0 and SD 1.

### Statistical analysis

Statistical analysis was carried out using Stata version 11. We inverse normalized circulating IPA levels as the metabolite concentration was not normally distributed. We imputed missing values using the minimum run-day measures. We assessed the association between the cardio-metabolic trait and circulating levels of IPA by using randomly mixed models adjusting for age, BMI and family relatedness. Linear mixed models were also employed to evaluate the associations between (i) circulating IPA levels and gut microbial diversity (Shannon, Simpson and CHAO1 indexes, number of observed OTUs), (ii) IPA and OTU, and (iii) IPA and taxa adjusting for age, BMI, technical covariates and multiple testing using Bonferroni correction.

As dietary fiber is a strong predictor of gut microbiome diversity,^24^ we further adjusted for fiber intake. To identify a set of OTUs independently associated with IPA, we fitted a stepwise regression, incorporating all significant OTUs together with age and BMI.

Using standard multiple linear regressions, we computed the proportion of the variance (R^2^) in IPA not explained by age, BMI and MAP that was explained by microbiome diversity, microbiome OTUs, genetics, and diet. Finally, we explored the direction of microbiome associations with IPA and compared them to those between the same microbes and visceral fat, a marker of metabolic syndrome. To do this we first ran linear regressions of the same taxa and OTUs with normalized visceral fat adjusting for age, BMI and technical covariates. We then further adjusted for IPA and ran in parallel the same regressions with IPA as the outcome variable adjusting for visceral fat.
