# Supplementary material for: Circulating levels of the anti-oxidant indoleproprionic acid are associated with higher gut microbiome diversity
Source: Gut Microbes. 2019 Apr 29;10(6):688–95. doi: 10.1080/19490976.2019.1586038 (PMC6866703; doi:10.1080/19490976.2019.1586038)
Supplement: Supplemental Material [file kgmi-10-06-1586038-s001.docx]

**Table S1. Association between cardiometabolic phenotypes and circulating IPA levels after adjusting for covariates.**

| Phenotype | Beta[95%CI ] | P |
| --- | --- | --- |
| eGFR | -0.69[-1.42;0.05] | 0.068 |
| Glucose | -0.08[-0.14;-0.02] | 0.012 |
| HOMA2-IR | -0.05[-0.08;-0.01] | 0.014 |
| PWV | -0.13[-025;-0.002] | 0.045 |
| T2D | -0.26[-0.58;0.06] | 0.114 |
| Visceral Fat | -0.06[-0.09;-0.02] | 0.002 |

**Table S2. Association between IPA levels and taxa adjusting for age, BMI and technical covariates and multiple testing using Bonferroni correction. The relative abundances of each of these taxa is also shown**

| Taxa Level | Taxa | Beta[95%CI] | P | P_adj | Relative abundance |
| --- | --- | --- | --- | --- | --- |
| Phyla | k__Bacteria;p__Tenericutes | 0.22[0.15;0.29] | 2.6E-10 | 9.63E-09 | 1.84% |
| Class | k__Bacteria;p__Tenericutes;c__Mollicutes | 0.21[0.14;0.28] | 8.82E-10 | 7.23E-08 | 1.78% |
| Family | k__Bacteria;p__Tenericutes;c__Mollicutes;o__RF39;f__ | 0.19[0.13;0.25] | 5.86E-10 | 1.83E-07 | 1.71% |
| Family | k__Bacteria;p__Firmicutes;c__Clostridia;o__Clostridiales;f__ | 0.13[0.07;0.19] | 2.32E-05 | 0.007263 | 9.88% |
| Order | k__Bacteria;p__Tenericutes;c__RF3;o__ML615J-28 | 0.19[0.13;0.25] | 5.86E-10 | 1.02E-07 | 1.71% |
| Genus | k__Bacteria;p__Tenericutes;c__Mollicutes;o__RF39;f__;g__ | 0.19[0.13;0.25] | 5.86E-10 | 4.09E-07 | 1.71% |
| Genus | k__Bacteria;p__Firmicutes;c__Clostridia;o__Clostridiales;f__Lachnospiraceae;g__[Ruminococcus] | -0.14[-0.2;-0.08] | 4.35E-06 | 0.003034 | 1.33% |
| Genus | k__Bacteria;p__Firmicutes;c__Clostridia;o__Clostridiales;f__;g__ | 0.13[0.07;0.19] | 2.32E-05 | 0.016174 | 9.87% |
| Genus | k__Bacteria;p__Firmicutes;c__Clostridia;o__Clostridiales;f__Lachnospiraceae;g__Blautia | -0.12[-0.18;-0.06] | 4.52E-05 | 0.031498 | 3.11% |
| Genus | k__Bacteria;p__Firmicutes;c__Erysipelotrichi;o__Erysipelotrichales;f__Erysipelotrichaceae;g__[Eubacterium] | -0.12[-0.18;-0.06] | 6.98E-05 | 0.048684 | 0.35% |
| Species | k__Bacteria;p__Firmicutes;c__Erysipelotrichi;o__Erysipelotrichales;f__Erysipelotrichaceae;g__[Eubacterium];s__dolichum | -0.2[-0.26;-0.14] | 1.28E-11 | 1.15E-08 | 0.03% |
| Species | k__Bacteria;p__Tenericutes;c__Mollicutes;o__RF39;f__;g__;s__ | 0.19[0.13;0.25] | 5.86E-10 | 5.23E-07 | 1.71% |
| Species | k__Bacteria;p__Firmicutes;c__Clostridia;o__Clostridiales;f__Lachnospiraceae;g__Coprococcus;Other | 0.18[0.12;0.23] | 4.8E-09 | 4.29E-06 | 0.46% |
| Species | k__Bacteria;p__Firmicutes;c__Clostridia;o__Clostridiales;f__Lachnospiraceae;g__Coprococcus;s__eutactus | 0.15[0.1;0.21] | 1.98E-07 | 0.000177 | 0.003% |
| Species | k__Bacteria;p__Firmicutes;c__Clostridia;o__Clostridiales;f__Lachnospiraceae;g__[Ruminococcus];s__ | -0.14[-0.2;-0.08] | 6.02E-06 | 0.005378 | 0.59% |
| Species | k__Bacteria;p__Firmicutes;c__Clostridia;o__Clostridiales;f__;g__;s__ | 0.13[0.07;0.19] | 2.32E-05 | 0.020722 | 9.88% |

**Table S3. Association between IPA levels and OTUs adjusting for age, BMI and technical covariates and multiple testing using Bonferroni correction.**

| Var | OTU | Beta[95%CI] | P |
| --- | --- | --- | --- |
| denovo753 | k__Bacteria; p__Firmicutes; c__Clostridia; o__Clostridiales; f__Lachnospiraceae; g__; s__ | -0.24[-0.29;-0.18] | 1.78E-15 |
| denovo165 | k__Bacteria; p__Firmicutes; c__Clostridia; o__Clostridiales; f__Lachnospiraceae; g__; s__ | -0.23[-0.29;-0.17] | 2.13E-14 |
| denovo57 | k__Bacteria; p__Firmicutes; c__Clostridia; o__Clostridiales; f__Lachnospiraceae; g__Lachnospira; s__ | 0.2[0.14;0.26] | 1.94E-11 |
| denovo90 | k__Bacteria; p__Firmicutes; c__Clostridia; o__Clostridiales; f__Lachnospiraceae; g__Coprococcus; s__ | 0.19[0.13;0.25] | 1.05E-10 |
| denovo329 | k__Bacteria; p__Firmicutes; c__Clostridia; o__Clostridiales; f__Lachnospiraceae; g__Dorea; s__ | -0.19[-0.25;-0.13] | 1.97E-10 |
| denovo43 | k__Bacteria; p__Firmicutes; c__Clostridia; o__Clostridiales; f__Lachnospiraceae; g__Coprococcus | 0.18[0.13;0.24] | 7.77E-10 |
| denovo469 | k__Bacteria; p__Firmicutes; c__Clostridia; o__Clostridiales; f__Ruminococcaceae; g__Faecalibacterium; s__prausnitzii | 0.18[0.12;0.24] | 2.94E-09 |
| denovo348 | k__Bacteria; p__Firmicutes; c__Clostridia; o__Clostridiales; f__Ruminococcaceae; g__; s__ | 0.17[0.11;0.23] | 2.91E-08 |
| denovo123 | k__Bacteria; p__Firmicutes; c__Clostridia; o__Clostridiales; f__Ruminococcaceae; g__; s__ | 0.17[0.11;0.23] | 3.06E-08 |
| denovo278 | k__Bacteria; p__Firmicutes; c__Erysipelotrichi; o__Erysipelotrichales; f__Erysipelotrichaceae; g__[Eubacterium]; s__dolichum | -0.17[-0.23;-0.11] | 3.23E-08 |
| denovo9 | k__Bacteria; p__Firmicutes; c__Clostridia; o__Clostridiales; f__; g__; s__ | 0.17[0.11;0.23] | 4.68E-08 |
| denovo59 | k__Bacteria; p__Firmicutes; c__Clostridia; o__Clostridiales; f__Ruminococcaceae; g__Oscillospira; s__ | 0.17[0.11;0.23] | 6.12E-08 |
| denovo72 | k__Bacteria; p__Firmicutes; c__Clostridia; o__Clostridiales; f__; g__; s__ | 0.16[0.1;0.22] | 9.37E-08 |
| denovo346 | k__Bacteria; p__Firmicutes; c__Clostridia; o__Clostridiales; f__; g__; s__ | 0.16[0.1;0.22] | 1.64E-07 |
| denovo355 | k__Bacteria; p__Firmicutes; c__Clostridia; o__Clostridiales; f__; g__; s__ | 0.16[0.1;0.21] | 2.28E-07 |
| denovo1143 | k__Bacteria; p__Firmicutes; c__Clostridia; o__Clostridiales; f__Lachnospiraceae; g__; s__ | 0.16[0.1;0.21] | 2.49E-07 |
| denovo109 | k__Bacteria; p__Firmicutes; c__Clostridia; o__Clostridiales; f__; g__; s__ | 0.15[0.1;0.21] | 3.29E-07 |
| denovo47 | k__Bacteria; p__Firmicutes; c__Clostridia; o__Clostridiales; f__Ruminococcaceae; g__; s__ | 0.15[0.09;0.21] | 3.84E-07 |
| denovo207 | k__Bacteria; p__Firmicutes; c__Clostridia; o__Clostridiales; f__Ruminococcaceae; g__; s__ | 0.15[0.09;0.21] | 4.53E-07 |
| denovo526 | k__Bacteria; p__Firmicutes; c__Clostridia; o__Clostridiales; f__Ruminococcaceae; g__Oscillospira; s__ | 0.15[0.09;0.21] | 7.34E-07 |
| denovo55 | k__Bacteria; p__Firmicutes; c__Clostridia; o__Clostridiales; f__Lachnospiraceae; g__[Ruminococcus]; s__ | -0.15[-0.21;-0.09] | 7.80E-07 |
| denovo495 | k__Bacteria; p__Firmicutes; c__Clostridia; o__Clostridiales; f__Lachnospiraceae; g__; s__ | -0.14[-0.2;-0.09] | 1.59E-06 |
| denovo367 | k__Bacteria; p__Firmicutes; c__Clostridia; o__Clostridiales; f__Lachnospiraceae; g__; s__ | -0.15[-0.21;-0.09] | 1.74E-06 |
| denovo163 | k__Bacteria; p__Firmicutes; c__Clostridia; o__Clostridiales; f__Ruminococcaceae; g__; s__ | 0.14[0.08;0.2] | 2.35E-06 |
| denovo1264 | k__Bacteria; p__Firmicutes; c__Clostridia; o__Clostridiales; f__Ruminococcaceae; g__; s__ | 0.14[0.08;0.2] | 2.45E-06 |
| denovo62 | k__Bacteria; p__Firmicutes; c__Clostridia; o__Clostridiales; f__; g__; s__ | 0.14[0.08;0.2] | 3.09E-06 |
| denovo1800 | k__Bacteria; p__Firmicutes; c__Clostridia; o__Clostridiales; f__Lachnospiraceae; g__; s__ | -0.14[-0.2;-0.08] | 3.81E-06 |
| denovo217 | k__Bacteria; p__Firmicutes; c__Clostridia; o__Clostridiales; f__Ruminococcaceae; g__Oscillospira; s__ | -0.14[-0.2;-0.08] | 4.89E-06 |
| denovo142 | k__Bacteria; p__Firmicutes; c__Clostridia; o__Clostridiales; f__Ruminococcaceae; g__Oscillospira; s__ | -0.14[-0.2;-0.08] | 5.16E-06 |
| denovo473 | k__Bacteria; p__Firmicutes; c__Clostridia; o__Clostridiales; f__Ruminococcaceae; g__; s__ | 0.14[0.08;0.2] | 6.23E-06 |
| denovo441 | k__Bacteria; p__Firmicutes; c__Clostridia; o__Clostridiales; f__Ruminococcaceae; g__; s__ | 0.14[0.08;0.2] | 6.23E-06 |
| denovo113 | k__Bacteria; p__Firmicutes; c__Clostridia; o__Clostridiales; f__Ruminococcaceae; g__Ruminococcus; s__ | 0.13[0.08;0.19] | 7.17E-06 |
| denovo41 | k__Bacteria; p__Tenericutes; c__Mollicutes; o__RF39; f__; g__; s__ | 0.13[0.08;0.19] | 7.44E-06 |
| denovo272 | k__Bacteria; p__Firmicutes; c__Clostridia; o__Clostridiales; f__Lachnospiraceae; g__; s__ | -0.13[-0.19;-0.08] | 7.64E-06 |
| denovo79 | k__Bacteria; p__Firmicutes; c__Clostridia; o__Clostridiales; f__Ruminococcaceae; g__; s__ | 0.13[0.08;0.19] | 8.69E-06 |
| denovo364 | k__Bacteria; p__Firmicutes; c__Clostridia; o__Clostridiales; f__Lachnospiraceae; g__[Ruminococcus]; s__ | -0.13[-0.19;-0.07] | 9.00E-06 |
| denovo808 | k__Bacteria; p__Firmicutes; c__Clostridia; o__Clostridiales; f__; g__; s__ | 0.13[0.07;0.19] | 1.12E-05 |
| denovo102 | k__Bacteria; p__Firmicutes; c__Clostridia; o__Clostridiales; f__Ruminococcaceae; g__Oscillospira; s__ | 0.13[0.07;0.19] | 1.55E-05 |
| denovo433 | k__Bacteria; p__Firmicutes; c__Clostridia; o__Clostridiales; f__Christensenellaceae; g__; s__ | 0.13[0.07;0.19] | 1.57E-05 |
| denovo277 | k__Bacteria; p__Firmicutes; c__Clostridia; o__Clostridiales; f__; g__; s__ | 0.13[0.07;0.19] | 2.43E-05 |
| denovo1174 | k__Bacteria; p__Firmicutes; c__Clostridia; o__Clostridiales; f__Ruminococcaceae; g__; s__ | 0.13[0.07;0.19] | 2.78E-05 |
| denovo13 | k__Bacteria; p__Firmicutes; c__Clostridia; o__Clostridiales; f__Lachnospiraceae; g__Blautia; s__ | -0.13[-0.19;-0.07] | 3.39E-05 |
| denovo12 | k__Bacteria; p__Firmicutes; c__Clostridia; o__Clostridiales; f__Ruminococcaceae; g__; s__ | 0.13[0.07;0.19] | 3.44E-05 |
| denovo527 | k__Bacteria; p__Firmicutes; c__Clostridia; o__Clostridiales; f__Ruminococcaceae; g__; s__ | 0.12[0.07;0.18] | 3.69E-05 |
| denovo344 | k__Bacteria; p__Firmicutes; c__Clostridia; o__Clostridiales; f__Ruminococcaceae; g__; s__ | 0.13[0.07;0.19] | 4.09E-05 |
| denovo578 | k__Bacteria; p__Firmicutes; c__Clostridia; o__Clostridiales; f__Ruminococcaceae; g__; s__ | 0.12[0.06;0.18] | 4.65E-05 |
| denovo1898 | k__Bacteria; p__Firmicutes; c__Clostridia; o__Clostridiales; f__Lachnospiraceae; g__; s__ | 0.12[0.06;0.18] | 4.76E-05 |
| denovo38 | k__Bacteria; p__Firmicutes; c__Clostridia; o__Clostridiales; f__Ruminococcaceae; g__Ruminococcus; s__ | 0.12[0.06;0.18] | 5.12E-05 |
| denovo383 | k__Bacteria; p__Firmicutes; c__Clostridia; o__Clostridiales; f__Lachnospiraceae; g__; s__ | 0.12[0.06;0.18] | 5.13E-05 |
| denovo129 | k__Bacteria; p__Firmicutes; c__Clostridia; o__Clostridiales; f__Lachnospiraceae; g__Lachnospira; s__ | 0.12[0.06;0.18] | 5.21E-05 |
| denovo70 | k__Bacteria; p__Firmicutes; c__Clostridia; o__Clostridiales; f__; g__; s__ | 0.12[0.06;0.18] | 5.77E-05 |
| denovo171 | k__Bacteria; p__Firmicutes; c__Clostridia; o__Clostridiales; f__Lachnospiraceae; g__; s__ | 0.12[0.06;0.18] | 0.000062 |
| denovo1315 | k__Bacteria; p__Firmicutes; c__Clostridia; o__Clostridiales; f__Lachnospiraceae; g__; s__ | 0.12[0.06;0.18] | 7.02E-05 |
| denovo691 | k__Bacteria; p__Firmicutes; c__Clostridia; o__Clostridiales; f__Dehalobacteriaceae; g__Dehalobacterium; s__ | 0.12[0.06;0.18] | 0.000074 |
| denovo958 | k__Bacteria; p__Firmicutes; c__Clostridia; o__Clostridiales; f__; g__; s__ | 0.12[0.06;0.18] | 7.58E-05 |
| denovo36 | k__Bacteria; p__Firmicutes; c__Clostridia; o__Clostridiales; f__; g__; s__ | 0.12[0.06;0.18] | 7.63E-05 |
